# The Dicistronic Nature of Picornavirus Genomes? Diverse Translation Mechanisms Enable the Expression of Accessory Proteins from Alternate Open Reading Frames

**DOI:** 10.3390/v18070797

**Published:** 2026-07-20

**Authors:** Christopher U. T. Hellen

**Affiliations:** Department of Cell Biology, SUNY Downstate Health Sciences University, Brooklyn, NY 11203, USA; christopher.hellen@downstate.edu

**Keywords:** picornavirus, IRES, translation initiation, termination–reinitiation, ribosomal scanning, programmed ribosomal frame-shifting

## Abstract

Members of the *Picornaviridae* family of viruses have single-stranded positive-sense RNA genomes, and are conventionally considered to contain a single open reading frame (ORF) preceded by a long 5′ untranslated region (UTR). The 5′UTR contains an internal ribosomal entry site (IRES) that mediates end-independent initiation of translation of ORF1, resulting in synthesis of a polyprotein that is proteolytically processed to yield the individual structural and nonstructural proteins. However, recent studies indicate that the dogma that picornavirus genomes contain a single ORF (ORF1) is an oversimplification, and that members of many picornavirus genera contain an additional ORF that is translated to yield an accessory protein that may influence pathogenicity and tissue specificity. This review summarizes the structural organization of picornavirus genomes that contain a second ORF. Most commonly, these ORFs overlap the 5′-end of ORF1, but they may also be wholly distinct from ORF1 or located entirely within it in a different reading frame. Ribosomal access to these alternate ORFs has been shown or is hypothesized to depend on a remarkable variety of non-canonical translation mechanisms, including ribosomal recruitment by a second IRES, leaky scanning after IRES-mediated internal ribosomal entry, cap-independent initiation, frame-shifting and termination–reinitiation.

## 1. Introduction

The size and structure of the genomes of positive sense RNA viruses are constrained by the packaging limit of virions and the potentially lethal combination of the low fidelity of replication coupled with a lack of proofreading. A further limitation is imposed by the canonical mechanism of protein synthesis in eukaryotes, which restricts translation to a single open reading frame (ORF). These constraints favor genome compression for RNA viruses. To maximize utilization of genomic coding potential, many viruses have evolved the ability to exploit non-canonical translation mechanisms that enable ribosomes to access additional ORFs and overlapping reading frames. These mechanisms include internal initiation, leaky scanning, reinitiation, programmed ribosomal frame-shifting and stop codon readthrough [1,2,3,4].

**Figure 1:**
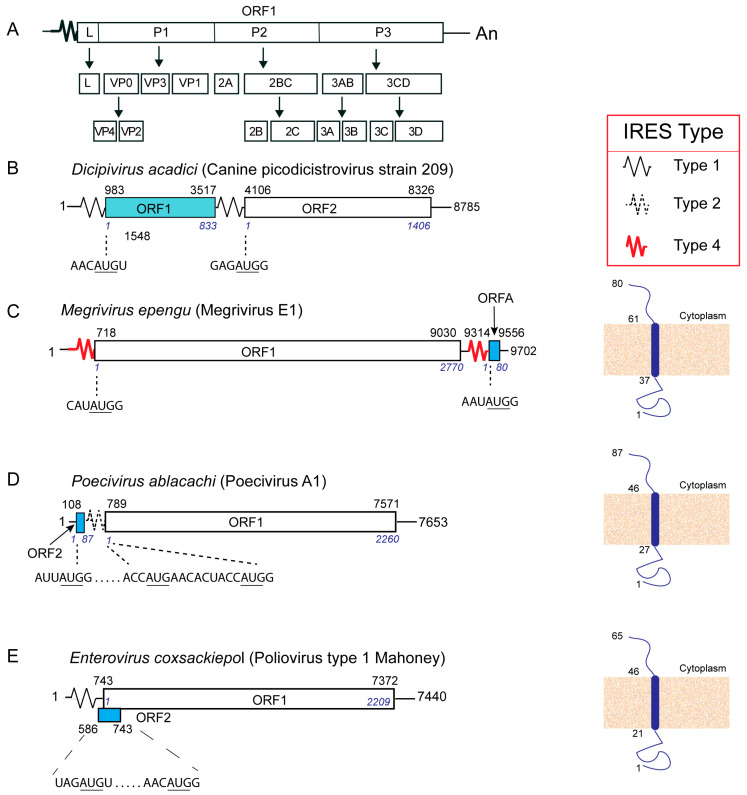
**Dicistronic genomes in picornaviruses.** Schematic representations of (**A**) a generic monocistronic picornavirus genome, showing the polyprotein-encoding ORF1, the individual proteins and their precursors derived by proteolytic cleavage of the polyprotein. A leader (L) protein is encoded by only a subset of picornaviruses. The L and the 2A proteins have proteolytic activity in only a subset of picornaviruses. (**B**, left panel) *Dicipivirus acadici* (Canine picodicistrovirus strain 209) (NCBI Ref. seq.: NC_021178.1), (**C**, left panel) *Megrivirus enpengu* (Megrivirus E1 isolate KGI-Bel-P5/2015) (GenBank: MF405436.1), (**D**) *Poecivirus ablacachi* (Poecivirus A1) (GenBank: KU977108.1) and (**E**, left panel) *Enterovirus coxsackiepol* (poliovirus type 1 Mahoney) (NCBI Ref. seq.: NC_002058.3), showing the relative locations of the polyprotein-encoding ORF1, in the case of *Dicipivirus acadici*, of ORF2, and (**C**–**E**) of the altORF ORF2, and the nucleotide context of the corresponding initiation codons (underlined). (**C**–**E**) Nucleotide and amino acid residues are numbered (plain text and italic, respectively), and different types of IRESs are represented by zigzag lines (as indicated in the inset key at top right). The structures of ORF2-encoded polypeptides (**B**–**D**, right-hand panels) are illustrated to show the *trans*-membrane domain, cytoplasmic element and non-cytoplasmic element, predicted (**C**,**D**) using the Phobius webserver [6], (**E**) as described [7] and numbered to indicate amino acid residues.

The *Picornaviridae* currently comprise 68 genera and numerous unclassified members. Here, species are named using the binomial nomenclature, together with common names if appropriate. Picornavirus genomes are conventionally described as mono-cistronic, encoding a single polyprotein that is cleaved into individual structural and nonstructural proteins by virus-encoded proteases [5] (Figure 1A). The capsid proteins VP4, VP2, VP3 and VP1 are derived from the N-terminal P1 region of the polyprotein, which in some viruses is preceded by a leader (L) protein. Nonstructural proteins 2A, 2B and 2C are cleaved from the central P2 region of the polyprotein, and the 3A, 3B, 3C (protease) and 3D (polymerase) proteins are derived from the C-terminal P3 region. The 2A protein is a chymotrypsin-like protease in many (but not all) picornaviruses. However, accumulating evidence indicates that the dogma that picornavirus genomes are monocistronic is an oversimplification, and that picornaviruses belonging to many genera utilize diverse mechanisms to enable translation of alternate open reading frames (altORFs). Translation of a single polyprotein limits the possibilities for control over the timing and stoichiometry of synthesis of proteins, whereas the presence of altORFs allows greater control over these aspects of gene expression. Here, I review this evolving topic, describe validated altORFs and suggest additional possible examples of picornavirus altORF translation.

## 2. The Canonical Mechanism of Translation in Eukaryotes

The cyclical process of eukaryotic translation consists of four stages: initiation, elongation, termination and post-termination recycling. The canonical initiation process [8] begins with separated 40S and 60S ribosomal subunits, derived by recycling of post-termination ribosomes or splitting of vacant 80S ribosomes. Aminoacylated initiator tRNA (Met-tRNA_i_^Met^) forms a ternary complex with eukaryotic initiation factor (eIF) 2 and GTP that binds to the 40S subunit with eIF1, eIF1A and the multi-subunit eIF3 to form a 43S preinitiation complex. Cellular mRNAs have a 5′-terminal m^7^G cap and 43S complexes are recruited to it by the cap-binding complex eIF4F, a heterotrimer that consists of eIF4E (the cap-binding subunit), eIF4A (an RNA helicase), and eIF4G (which binds to eIF4E, eIF4A, and eIF3). The 43S complex then scans downstream in a process that integrates ribosomal movement, unwinding of secondary structure in the 5′ untranslated region (5′UTR) of the mRNA and monitoring of the 5′UTR to identify the initiation codon, which is the first AUG triplet in a ‘good’ context (e.g., **A**CCAUG**G**), defined by the presence of purine residues at −3 and +4 positions (bold) relative to the AUG codon (underlined). AUG codons in a suboptimal context are predominantly bypassed by ‘leaky scanning’, leading to initiation further downstream. Scanning on structured 5′UTRs also requires the DExH-box helicase DHX29 [9]. Recognition of context nucleotides involves establishment of specific interactions between them and eIF1A, eIF2 and the 18S ribosomal RNA (rRNA) constituent of the 40S subunit [10,11]. When the scanning complex encounters a favorable AUG codon, hydrolysis of eIF2-bound GTP is induced by eIF5, eIF1 is displaced and initiator tRNA is released into the ribosomal peptidyl (P) site, resulting in formation of a 48S complex. The scanning-competence of 43S complexes is dependent on eIF1, which also enforces the fidelity of initiation codon selection [8,12,13]. eIF1 induces a scanning-permissive conformation of the 43S complex and maintains initiator tRNA in a metastable state that is permissive for scanning and initiation codon inspection and that is adjusted following displacement of eIF1, resulting in formation of the 48S complex with established codon–anticodon base-pairing [14]. eIF5B induces reorientation of initiator tRNA on the 40S subunit and the joining of a 60S subunit, thereby promoting dissociation of factors from the interface surface of the 40S subunit and formation of an 80S ribosome [8].

Elongation involves cycles of delivery of cognate aminoacyl-tRNA to the ribosome by eukaryotic elongation factor (eEF) 1A•GTP to decode the mRNA codon in the ribosomal aminoacyl (A) site, release of eEF1A•GDP, peptide bond formation, and translocation promoted by eEF2 of the base-paired mRNA and peptidyl-tRNA from A to P sites [15]. Eukaryote-specific elements of the ribosome, tRNAs and eEF2 prevent slippage of the translational reading frame [16]. However, specific sequence motifs and structures in the coding region can induce programmed frame-shifting so that ribosomes continue translation in a different reading frame [4]. The 3′-end of an ORF is marked by a stop codon, and its entry into the A site signals the termination of translation by eukaryotic release factor (eRF) 1 and eRF3•GTP [17]. eRF1 recognizes the stop codon, which triggers hydrolysis of eRF3-bound GTP and release of eRF3•GDP, followed by conformational changes in eRF1 that enable it to enter the ribosomal peptidyl transferase center, triggering cleavage of peptidyl-tRNA and release of the completed polypeptide [18]. In the final stage of translation, the post-termination ribosomal complex is split by ABCE1, releasing the 60S subunit [19]. The 40S subunit remains bound to mRNA and deacylated tRNA, which are released via redundant mechanisms involving the factors eIF2D, or MCTS1/DENR, or eIF1, eIF1A and eIF3.

Three of these four stages in the translation process are or have the potential to be subverted to enable translation of altORFs in picornavirus genomes in addition to the principal ORF.

## 3. Initiation of Picornavirus Translation by Internal Ribosomal Entry

Picornavirus infections commonly shut off the canonical initiation process, for example by viral protease-mediated cleavage of eIF4G’s N-terminal eIF4E-binding domain from its eIF4A- and eIF3-binding domains, and by activation of the regulatory 4E-binding protein, which sequesters eIF4E [20]. Both mechanisms impair eIF4F’s functions in binding to the capped 5′-end of mRNA and in recruiting the 43S complex to it. The 5′-terminus of picornavirus genomic RNAs is not capped, and is instead covalently attached to 3B (also known as the genome-linked protein VPg), which is cleaved from the viral RNA after its release from virions in infected cells. Initiation of translation on picornavirus mRNAs is therefore cap-independent. This process is mediated by an internal ribosomal entry site (IRES) that is usually located between structured elements at the 5′-end of the 5′UTR that function during replication and the initiation codon of the polyprotein-encoding ORF1. IRESs are classified into six major groups (types 1–6) on the basis of sequence and structure (nomenclature according to [21]). Type 6 IRESs occur predominantly in dicistroviruses and will not be discussed here because they have not been identified in picornaviruses. I will not focus on details of the structure and mechanism of action of IRES types 1–5 here, but instead on their capacity to direct translation of altORFs.

Type 1 IRESs were discovered in *Enterovirus coxsackiepol* (poliovirus) in the genus *Enterovirus* [22], and also occur in the *Boosepivirus*, *Crohivirus*, *Dicipivirus* and *Harkavirus* genera [21,23,24]. Canonical type 1 IRESs are ~450 nt long and contain five domains (II–VI). Domain VI overlaps a Y*n*-X*m*-AUG motif (comprising a pyrimidine-rich tract (*n* = 8–10) separated by a spacer (*m* = 18–20) from an AUG triplet) at the 3′-border of the IRES [25] which in different enterovirus genomes is ~30 nt to ~160 nt upstream of the initiation codon of the principal ORF. The Y*n* and AUG elements of this motif are positive determinants of the efficiency of initiation [7,25,26]. Internal ribosomal entry on type 1 IRESs is eIF4E-independent but otherwise requires the full complement of eIFs [27] and depends on binding of the central (eIF4A-binding) domain of eIF4G and eIF4A to domain V [28]. The 43S complex binds to the IRES immediately upstream of the AUG triplet of the Y*n*-X*m*-AUG motif [29,30] and scans downstream to the initiation codon in an eIF1-dependent manner [27]. Type 1 IRES function is also dependent on one or more IRES *trans*-acting factors (ITAFs), which are cellular RNA-binding proteins that are not involved in the canonical initiation process.

Type 2 IRESs occur in many picornavirus genera, and are exemplified by *Cardiovirus rueckerti* (encephalomyocarditis virus (EMCV)) (genus *Cardiovirus*) [31] and *Aphthovirus vesiculae* (foot-and-mouth disease virus (FMDV)) (genus *Aphthovirus*) [32]. They comprise five domains (H–L), are ~450 nt long and also have a Y*n*-X*m*-AUG motif at their 3′-border. Initiation requires the full set of canonical eIFs except for eIF4E [12,33], and depends on binding of eIF4G to the J-K domain [34,35,36] and on interaction of the apical region of domain I with initiator tRNA and the 40S subunit [37]. The 43S complex binds the viral mRNA at or immediately upstream of the AUG codon of the Y*n*-X*m*-AUG motif [38] but scans downstream if subunit joining is delayed on the EMCV IRES [37,39,40] or, in an eIF1-dependent manner, bypasses the AUG1 codon of the Y*n*-X*m*-AUG motif of the FMDV IRES and scans downstream to AUG2, 84 nt downstream of AUG1 [41,42,43,44]. *Aphthovirus burrowsi* (genus *Aphthovirus*) and *Erbovirus aequirhi* (genus *Erbovirus*) similarly contain multiple functional in-frame AUG codons at and downstream of the Y*n*-X*m*-AUG motif, and elimination of upstream AUG codons increased initiation at downstream codons, consistent with ribosomal scanning after internal ribosomal entry [45,46]. The initiation codon for the polyprotein-encoding ORF1 is far downstream of the Y*n*-X*m*-AUG motif in numerous picornaviruses, such as *Parechovirus efalco* (Genbank: NC_035779.1) [47] and *Sicinivirus ahiberni* (GenBank: MN873048.1), which are therefore likely also reached by scanning after internal ribosomal entry.

Type 3 IRESs occur in *Hepatovirus ahepa* (Hepatitis A virus (HAV)) and other species in the genera *Hepatovirus* [48], *Crahelivirus* and *Gruhelivirus* [49]. The HAV IRES has a Y*n*-X*m*-AUG motif at its 3′border and initiation can occur at AUG_735_ and at the nearby AUG_741_ [50]. Initiation on this IRES is incompletely characterized, but it requires canonical initiation factors, including eIF1A, eIF3 and eIF4B ([51] and references therein), and, exceptionally, is strongly dependent on eIF4E and intact eIF4G [52]. eIF4G is thought to bind to domain V [53,54]; eIF4E enhances binding of eIF4G to the IRES and eIF4A’s helicase activity [55] and anchors the cellular PDGFA-associated protein 1, which also interacts with eIF1A [51].

Type 4 IRESs were discovered in hepatitis C virus and other members of *Flaviviridae*, but following the report of a type 4 IRES in the 5′UTR of *Teschovirus asilesi* (genus *Teschovirus*) [56], they have subsequently been identified in almost half of all picornavirus genera [21,57,58,59,60]. They are 235–400 nt long, have a common core structure centered on a double pseudoknot and a branching hairpin with an apical GGG loop motif [59,60], and function without the involvement of eIFs 1, 4A, 4B or 4F by direct factor-independent interaction with the 40S ribosomal subunit [56,61,62,63].

Type 5 IRESs occur in members of the genera *Kobuvirus*, *Oscivirus*, *Passerivirus* and *Salivirus*, and appear to be chimeric, containing six conserved domains (G–L). Domains H and J resemble domains III and IV of type 1 IRESs, domain K contains motifs identical to those in domain J of type 2 IRESs and domain L overlaps a Y*n*-X*m*-AUG motif [64,65,66]. Initiation on these IRESs requires DHX29 and the complete set of canonical eIFs except eIF4E [64,65]. Notably, the separation between the conserved core of these IRESs and the principal initiation codon for the polyprotein ORF is highly variable, and similarly to type 1 and type 2 IRESs, initiation on type 5 IRESs can therefore also involve scanning after internal ribosomal entry [65,67].

## 4. IRES-Mediated Initiation of Translation of Dicistronic Picornavirus Genomes

Although most picornaviruses contain a single long ORF that encodes a large polyprotein (Figure 1A), this ORF is split in several naturally occurring dicistronic picornaviruses. They contain a second IRES, located in the intergenic region (IGR) between ORFs that encode structural and nonstructural protein precursors, respectively. A second group of picornaviruses that have a second IRES contain a unitary principal ORF, and the IRES is located beyond its 3′border, positioned to promote translation of a small ORF that encodes an accessory protein.

Members of the genus *Picodicistrovirus* such as canine picodicistrovirus (CPDV) have dicistronic genomes (Figure 1B): the P1 (capsid protein precursor) and P2-P3 (nonstructural protein precursor) ORFs are both preceded by a divergent type 1 IRES [23,68,69]. The genomes of a sister lineage, exemplified by Wenzhou shrew picornavirus 1 (GenBank: OQ716059.1), have a similar dicistronic structure [70]. These divergent IRESs are most similar to canonical type 1 IRESs in their 3′-terminal regions, which contain a domain IV-like structure with a characteristically conserved apical GNRA tetraloop, a domain related to domain V and (in the intergenic region (IGR) IRES) a Y*n*-X*m*-AUG motif that overlaps domain VI. There are significant relative insertions and deletions in the 5′-terminal IRESs, consistent with a history of multiple recombination, which likely also accounts for the divided coding region and the presence of related IRESs in the 5′UTR and the IGR [69]. The CPDV 5′UTR and IGR IRESs are both active in canine cells; the former has greater activity in some circumstances, suggesting that these IRESs may drive different levels of expression of capsid and nonstructural proteins [23]. In vitro reconstitution of initiation revealed that 48S complex formation on the CPDV 5′UTR IRES resembled initiation on type 1 IRESs in requiring the complete set of canonical initiation factors (except for eIF4E) and the ITAF poly(C)-binding protein 2 (PCBP2), in involving comparable interactions of the IRES with eIF4G/eIF4A and with PCBP2, and in being dependent on the GNRA tetraloop [71,72]. Significantly, initiation on the CPDV 5′-terminal IRES involves eIF1-dependent scanning for 30 nt or more after attachment to the IRES to the initiation codon [71].

Whereas the presence of an IRES between P1 and P2-P3 polyprotein-encoding ORFs in picodicistrovirus genomes potentially enables differential regulation of the timing and stoichiometry of synthesis of nonstructural and capsid proteins that are required at different stages of the viral replication cycle, the presence of a second IRES in the genomes of members of *Megrivirus epengu* (genus *Megrivirus*) enables translation of an accessory protein encoded by ORF2, downstream of the polyprotein-encoding ORF1 (Figure 1C, left panel). Megriviruses have a type 4 IRES in the 5′UTR in which domain 3 is exceptionally elongated [59]. The intergenic region between ORF1 and ORF2 in *Megrivirus epengu* ranges in size from 267 to 283 nt (e.g., [73,74,75]) and bioinformatic analysis led to the identification of a compact conserved type 4 IRES in this region [76]. Domain 2 of this IRES is unrelated to the corresponding element in other type 4 IRESs, and domain 3 is severely truncated. This IRES exploits the same initiation mechanism as conventional type 4 IRESs [61,62,63] by binding directly to the 40S subunit and to eIF3 [76]. However, its activity (in vitro) is lower than that of the 5′-terminal IRES, and it therefore has the potential to promote translation of ORF2 independently of ORF1 and likely at a lower level. ORF2 in different megriviruses encodes 71–83 amino acid long polypeptides that are likely *trans*-membrane proteins (Figure 1C, right panel) [7,76,77]. Remarkably, related polypeptides are encoded by altORFs in genomes of other species of *Megrivirus*, albeit not in association with an adjacent IRES, and in *Poecivirus* genomes [7,77,78,79]. Initiation mechanisms potentially leading to translation of these proteins are discussed below. Bioinformatic analyses [77] suggested that they may be cellular fusion-associated transmembrane (FAST) proteins [80], which are simple fusogens that contain a single *trans*-membrane domain and short extracellular and cytoplasmic domains, are commonly N-terminally myristoylated and promote cell–cell fusion. By analogy with the function of the well-characterized reovirus FAST proteins, these picornavirus proteins may facilitate cell-to-cell transmission of viruses.

## 5. End-Dependent Initiation of an Alternative ORF on Poecivirus Genomic mRNA?

Poecivirus genomes encode a single large polyprotein [81,82], preceded by a type 2 IRES that is related to those of members of the genera *Avisivirus* and *Sicinivirus* of *Picornaviridae* and of several avian caliciviruses [67,83]. A 272–312 nt long ORF2 located near the 5′ end of the genome upstream of the IRES (Figure 1D, left panel) encodes a highly conserved putative FAST Protein (Figure 1D, right panel) [78]. It is possible that this ORF is translated following cap-independent but end-dependent initiation: initiation complexes can scan through stable secondary structure (e.g., [9]) and could therefore potentially scan from the 5′-end of poecivirus genomes through the first ~100 nt of the 5′UTR to the ORF2 initiation codons (AUG_108_ in *Poecivirus ablacachi* (strain BCCH-449: GenBank NC_055108.1) and AUG_107_ in strain RBNU-929 (GenBank: MN944596.1)). In light of a report that IRESs can stimulate translation of upstream ORFs [84], one could speculate that the type 2 IRES downstream of ORF2 might stimulate translation of this protein.

## 6. Translation of Overlapping ORFs Following IRES-Mediated Initiation

The AUG triplet of the Y*n*-X*m*-AUG motif at the 3′-border of type 1 IRESs (poliovirus AUG_586_ and its equivalent in other enteroviruses) is an important determinant of IRES function [7,26], but it also supports robust initiation of translation in vitro if its weak context is improved, leading to translation of a 65-codon-long ORF2 that overlaps AUG_743_, the initiation codon for ORF1, which encodes the poliovirus polyprotein [25,27] (Figure 1E, left panel). Ribosomal entry onto the viral mRNA likely occurs on the Y*n*-X*m*-AUG motif [30] and ribosomes then scan downstream, either arresting at this AUG codon or scanning past it to the ORF1 initiation codon. Many enterovirus genomes contain ORF2 sequences that encode related 56–76-a.a.-long polypeptides that are predicted to contain a *trans*-membrane domain (Figure 1E, right panel) and that similarly overlap ORF1 [7,25,30,85]. Notably, ORF2 occurs in enteric but not in respiratory enteroviruses [25,84]. Although early studies using cell-free translation extracts had suggested that initiation occurred on two AUG codons in the poliovirus genome, leading to synthesis of an unidentified 5–10 kDa polypeptide and to the structural protein precursor [86,87], the ORF2-encoded polypeptide had escaped detection in enterovirus-infected cells, possibly due to its small size. However, it was observed by Western blotting of native or tagged forms in cells infected by echovirus 7, enterovirus 71 and poliovirus [7,85]. Ablation of ORF2 compromised replication of echovirus 7 in human intestinal cells [7] and replication of enterovirus 71 in human intestinal and murine neuronal cells [85]. Ectopically expressed ORF2 from heterologous enteroviruses enabled the replication in human intestinal cells of respiratory enteroviruses that naturally lack ORF2 (e.g., Enterovirus D68) or in which it has been inactivated, and this activity was dependent on the putative *trans*-membrane domain [85]. The ORF2 product promotes virus release from enteric cells [7,85].

**Figure 2:**
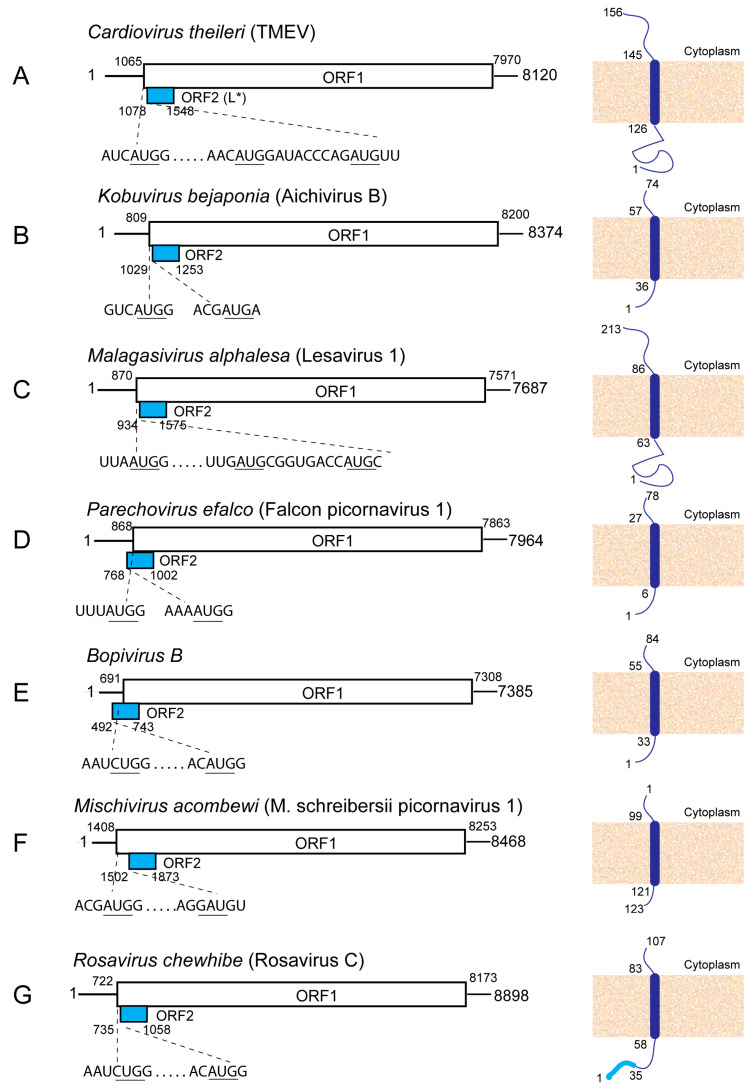
**Overlapping ORFs in picornavirus genomes that encode putative single-pass *trans*-membrane proteins.** Schematic representations of the genomes of (**A**) *Cardiovirus theileri* (Theiler’s murine encephalomyelitis virus strain DA; GenBank: JX443418), (**B**) *Kobuvirus bejaponia* (Aichivirus B strain U-1; NCBI Ref seq. NC_004421.1), (**C**) *Malagasivirus alphalesa* (Lesavirus 1 isolate Mis 101308/2012; GenBank: KM396707.1), (**D**) *Parechovirus efalco* (Falcon picornavirus 1; NCBI Ref. seq. NC_035779.1), (**E**) *Bopivirus B* (Bopivirus p. strain ovine/TB14.2010-Hun; GenBank: MW298057.1), (**F**) *Mischivirus acombewi* (Miniopterus schreibersii picornavirus 1; GenBank: JQ814851.1) and (**G**) *Rosavirus chewhibe* (Rosavirus C strain RASK8F; NCBI Ref. seq. NC_075425.1) showing the relative locations of the polyprotein-encoding ORF1 and the overlapping ORF2, the 5′UTRs and 3′UTRs (solid lines), and the nucleotide context of initiation codons. Dashed lines indicate the positions of ORF1 and ORF2 initiation codons. (**A**–**F**, right-hand panels) ORF2-encoded polypeptides, illustrated to show signal sequences (light blue), the *trans*-membrane domain (dark blue), cytoplasmic element and non-cytoplasmic element, predicted using the Phobius webserver [6].

The DA strain of Theiler’s murine encephalomyelitis virus (TMEV; *Cardiovirus theileri*) is a member of the genus *Cardiovirus*. Its type 2 IRES promotes translation of the ~18 kDa L* protein from the good-context AUG_1078_ and from AUG_1090_, both located downstream of AUG_1065_, the initiation codon for the polyprotein (Figure 2A, left panel), likely as a result of leaky scanning after ribosomal entry onto the Y*n*-X*m*-AUG motif [88,89]. The L* ORF is present in other TMEV strains [90], although in some, initiation occurs at near-cognate ACG codons located at equivalent positions rather than at AUG codons [91,92]. It is predicted to encode a *trans*-membrane protein (Figure 2A, right panel), and related proteins are translated from an overlapping ORF in the genomes of other *Cardiovirus* species, including *C. ranori****,*** *C. rudhira* and *C. dhusarah* [7]. A similar L* polypeptide is encoded by some Saffold viruses (which are also members of *C. theileri* (e.g., NC_009448.1)), and its translation likely also initiates at an ACG (Thr) codon, but in most of them, this altORF is interrupted by a stop codon that truncates the encoded protein before the predicted *trans*-membrane domain is translated [93]. L* determines the ability of the DA and related strains to establish a persistent infection in the central nervous system of mice [91,94]. It accumulates in the mitochondrial outer membrane [95], and binds to RNAse L, an important anti-viral innate immune effector, thereby preventing its interaction with and activation by 2′, 5′-oligoadenylates [96,97]. L* is therefore a determinant of pathogenesis, and remarkably, of species specificity, since L* from the (murine) DA strain of TMEV does not inhibit RNAse L from chickens, humans and numerous other mammals, even including rats [96].

**Figure 3:**
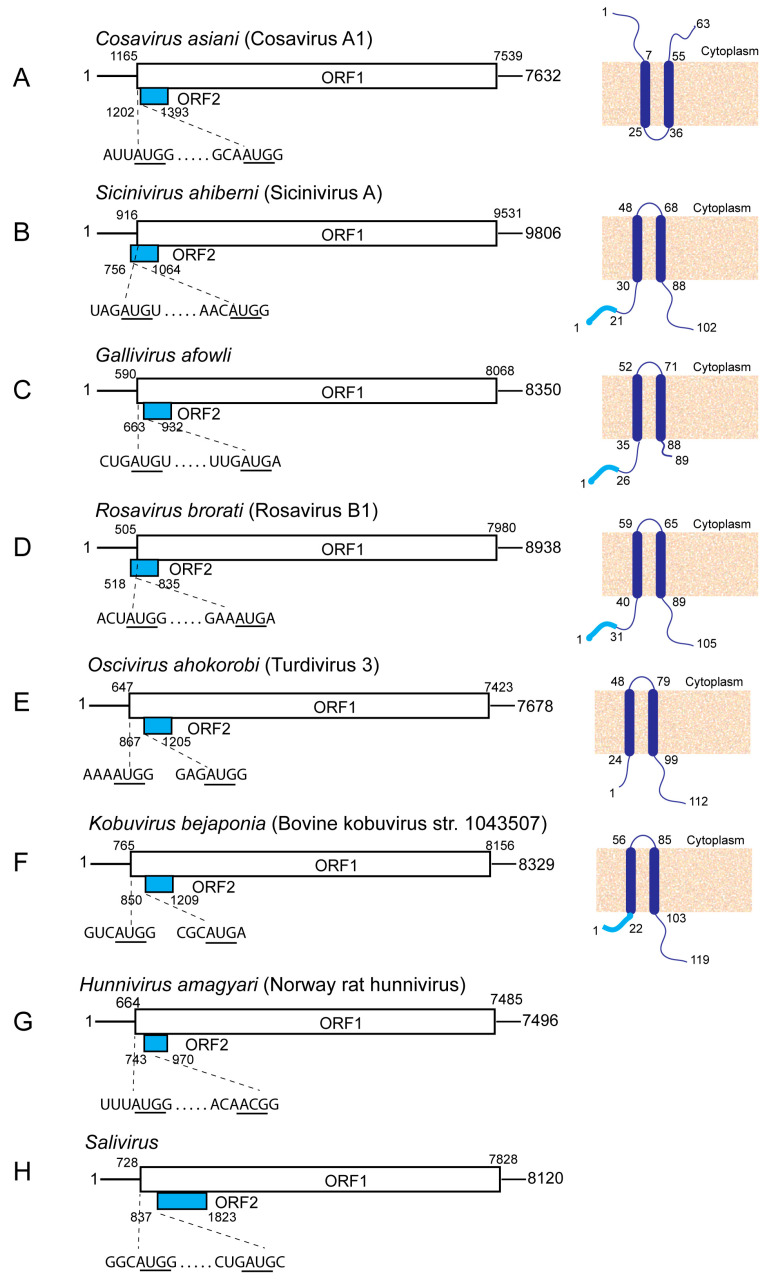
**Overlapping ORFs in picornavirus genomes.** Schematic representations of the genomes of (**A**) *Cosavirus asiani* (Cosavirus A1 strain HCoSV-A1; GenBank: FJ438902.1), (**B**) *Sicinivirus ahiberni* (Sicinivirus A isolate GA/1479/2004; GenBank: MN873048.1), (**C**) *Gallivirus afowli* (Gallivirus sp. isolate GALL/PB18-HII34/Switzerland/2019; GenBank: OM469266.1), (**D**) *Rosavirus brorati* (Rosavirus B1 strain RNCW0602091R; GenBank: KX783423.1), (**E**) *Oscivirus ahorkorobi* (Turdivirus 3 strain 10878; GenBank GU182410.1), (**F**) *Kobuvirus bejaponia* (Bovine kobuvirus strain 1043507 (GenBank: MW605074.1)), (**G**) *Hunnivirus amagyari* (Norway rat hunnivirus isolate NrHuV/NYC-E21) (GenBank: KJ950971.1) and (**H**) *Salivirus* FHB (Genbank: NC_025114.1), showing the relative locations of the polyprotein-encoding ORF1 and the overlapping ORF2, the 5′UTRs and 3′UTRs (solid lines), the nucleotide context of initiation codons. Dashed lines indicate the positions of ORF1 and ORF2 initiation codons. (**A**–**E**, right-hand panels) ORF2-encoded polypeptides, illustrated to show signal sequences (light blue), the *trans*-membrane domain (dark blue), cytoplasmic element and non-cytoplasmic element, predicted using the Phobius webserver [6].

Analysis of the coding potential of members of numerous other picornavirus genera indicates that they also contain altORFs that overlap the 5′-terminal region of the polyprotein-encoding ORF1. These genera include *Cosavirus*, *Hunnivirus*, *Malagasivirus*, *Mischivirus*, *Oscivirus*, *Rosavirus*, *Sicinivirus* [7], *Bopivirus, Gallivirus*, *Parechovirus*, *Kobuvirus* and *Salivirus* (Figure 2 and Figure 3). The polypeptides encoded by these altORFs fall into various categories, including proteins with predicted single- and multiple-pass *trans*-membrane domains as well as unclassified proteins (Figure 2 and Figure 3). Expression of these altORFs in the context of viral infection has not been confirmed, but several have been found to be subject to strong purifying selection, indicating that they encode functional proteins [7]. The level of expression of these altORFs in infected cells may be low, because their initiation codons are often in a suboptimal nucleotide context, and some lie downstream of the ORF1 initiation codon. Moreover, as noted previously, the altORFs in genomes of *Hunnivirus amagyari* [7] and *Bopivirus B* [98] initiate at conserved near-cognate ACG and CUG initiation codons respectively (Figure 2E and Figure 3G).

The presence of altORFs in members of some virus species is sporadic. For example, the 123-a.a.-long polypeptide encoded by the altORF in Bopivirus sp. strain deer/VIC82-2020/AUS (Genbank: MZ436972.1) shares 51–63% identity with polypeptides encoded by similarly positioned sequences in other bopivirus genomes (Genbank: NC_026249.1 and MW298059.1) which are, however, interrupted by one and two stop codons, respectively. Similarly, a 210 nt long element in parechovirus sp. isolate SQ/N.fuscus/Pare (Genbank PX095822.1) that is positioned similarly to the *Parechovirus falco* ORF2 (Figure 2D) would encode a related polypeptide to this altORF product, but is interrupted by three stop codons. Whereas *Kobuvirus bejaponia* (Bovine kobuvirus strain 1043507 (GenBank: MW605074.1)) encodes a 119-a.a.-long polypeptide with two predicted *trans*-membrane domains (Figure 3F), there are 5′-terminal substitutions in the equivalent sequence in *Kobuvirus bejaponia* (Aichivirus B strain U-1; NCBI Ref seq. NC_004421.1) that lead to loss of the initiation codon and gain of a stop codon, so that the residual ORF encodes a truncated 74-a.a.-long polypeptide that is nevertheless 80% identical to the longer equivalent (Figure 2B). Notably, these altORFs all occupy a genomic location that is a known hotspot for recombination [21,99]. Their sporadic appearance and apparent mutational inactivation in related viruses are consistent with a dynamic process of repeated acquisition of altORFs by recombination, followed either by retention and optimization or, if they do not confer any selective advantage on these viruses, by degeneration and loss.

ORF2 in many these viral genomes occurs downstream of type 2 or type 5 IRESs. As described above, initiation mediated by type 2 IRESs can involve scanning after ribosomal attachment, and as a result, some initiation complexes bypass an initiation codon near the landing site and scan downstream at a level that depends on the identity of the IRES, the nucleotide context of the initiation codon and the intracellular environment [41,43,100]. Type 5 IRESs are commonly separated from the polyprotein initiation codon by large variable spacers, suggesting that initiation mediated by them also involves scanning [64]. An IRES-initiated leaky scanning mechanism would allow regulation of ORF2 translation both with respect to the stoichiometry and the time of appearance of its product relative to the ORF1-encoded polyprotein. The level of ORF2 translation in genomes in which it overlaps ORF1 may be low early infection, because the initiation codon is usually in a poor nucleotide context, is commonly positioned downstream of the ORF1 polyprotein initiation codon, and in the case of genus *Oscivirus*, *Hunnivirus, Bopivirus* and some *Cardiovirus* genomes, initiates at near-cognate ACG or CUG codons [91,92] or is likely to do so [7]. However, the cytoplasmic environment is progressively altered during picornavirus infection in a manner that could affect ORF2 translation, for example by viral protease-mediated cleavage of some initiation factors (including eIF4A, eIF4G, eIF5B and the poly(A)-binding protein) and ITAFs (e.g., PCBP2), altered subcellular localization of initiation factors and ITAFs, activation of translational regulators and induced changes in intracellular ionic conditions [101,102,103].

## 7. Programmed Ribosomal Frame-Shifting During the Elongation Phase of Cardiovirus Translation

**Figure 4:**
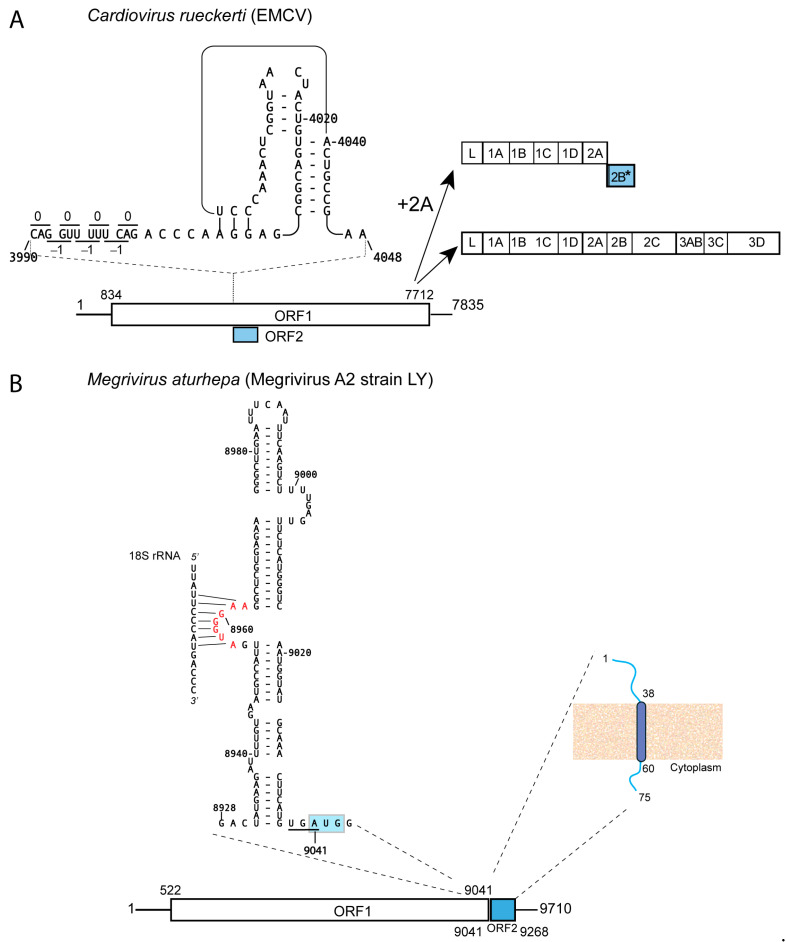
**RNA structure-mediated initiation/reinitiation of translation of picornavirus altORFs.** (**A**) The cardiovirus ribosomal frame-shifting element, showing the sequence and structure of this element in the EMCV genome (GenBank: NC_001479.1), its position relative to the G_GUU_UUU slippery sequence and the codons in the 0 and −1 reading frames. Binding of the viral 2A protein to this pseudoknot structure shifts ribosomes from translation of the polyprotein-encoding ORF1 to translation of the 2B* protein in the −1 reading frame followed by termination of translation, as indicated b the arrows. (**B**) The sequence and structure of the putative termination upstream ribosome binding site (TURBS) element in the genome of *Megrivirus aturhepa* (NCBI Ref. seq.: NC_024120.1) and its position immediately upstream of the overlapping ORF1 termination codon and the ORF2 initiation codon (indicated by a light blue shaded box). The conserved motif 1 sequence AUGGGAA (red font) can base-pair to an element of helix 26 of 18S rRNA, anchoring the post-termination ribosome prior to reinitiation. The ORF2-encoded polypeptide is illustrated to show the *trans*-membrane domain (dark blue), cytoplasmic element and non-cytoplasmic element, predicted using the Phobius webserver [6]. Dashed lines indicate (**A**,**B**) the positions of RNA elements and (**B**) link ORF2 to the FAST protein that it is predicted to encode.

The genomes of *Cardiovirus rueckerti* (EMCV) and other members of the *Cardiovirus* genus contain a ~120-codon-long region of elevated synonymous site conservation immediately after the 2A/2B junction in the −1 reading frame relative to the polyprotein ORF1 [104]. Frame-shifting occurs at a G-GUU-GUU ‘slippery’ sequence and is promoted with high (~70%) efficiency by an RNP complex that is formed by binding of the viral 2A protein to an adjacent downstream RNA structure (Figure 4A), likely resulting in a conformational change in it from a hairpin to a pseudoknot [105,106]. Frame-shifting on the EMCV genomic mRNA results in synthesis of a novel 129-a.a.-long *trans*-frame protein (2B*) [104], after which translation terminates. 2B* enhances virus release via caspase-3-mediated and caspase-3-independent cell lysis pathways [107] and binds to members of the 14-3-3 family of scaffold proteins, likely disrupting innate immune signaling [108]. Loss of 2B* leads to reduced viral plaque size and impaired lytic virus release [104,109]. The requirement of the 2A protein for effective frame-shifting leads to temporal control over translation: the viral polyprotein, including the nonstructural proteins that are derived by proteolytic cleavage of the P2 and P3 regions of the polyprotein, are translated early in infection, and as 2A accumulates, it promotes translation of 2B* followed by termination of translation. Therefore, 2A acts as a switch that downregulates translation of replication proteins so that the translation apparatus becomes dedicated to synthesis of structural proteins [109].

Frame-shifting elements of the same type occur in *Cardiovirus theileri* (TMEV), in *C. rudhira* and in *C. saffoldi* (e.g., [104]). In *C. theileri*, this element promotes frame-shifting at up to 82% efficiency [110]. The 2B* transframe polypeptides derived from these genomes would be only 14 a.a. long and are therefore likely nonfunctional. However, the 2A-mediated induction of translation termination before the synthesis of replicative proteins is important, because it enforces a switch to preferential translation of structural proteins late in infection. Impairment of frame-shifting resulted in TMEV having a small plaque phenotype and reduced growth kinetics, indicating the likely importance of this switch for viral replication [110,111].

## 8. Termination–Reinitiation Elements in Megrivirus Genomes

Numerous megrivirus genomic sequences that contain a FAST protein-encoding ORF2 [7,76,77] lack obvious IRES-like sequences between it and the polyprotein-encoding ORF1, and therefore likely exploit alternative mechanisms for initiation of ORF2 translation. In two groups of these megrivirus genomes, the ORF1 termination codon (underlined) overlaps the ORF2 initiation codon (bold) (UA**A****UG** or UG**A****UG**) or is separated from it by two or three nucleotides (UAA-2/3nt-**AUG** (Figure 4B) or, less commonly, UAG-2/3nt-**AUG**). This configuration of stop and start codons suggests that these ORF2 entities could be translated by a termination–reinitiation mechanism. The best-characterized mechanism of this type occurs on bicistronic subgenomic calicivirus mRNAs: translation of the minor capsid protein from the second ORF is mediated by a ~40–80 nt long TURBS (termination upstream ribosome binding site) immediately upstream of the restart AUG [112]. This structured RNA contains an essential conserved motif 1, whose AUGGGA core is complementary to the apical loop of helix 26 of 18S rRNA and which tethers the post-termination 40S ribosomal subunit to the stop/restart region, and the species-specific motif 2, which base-pairs to the complementary motif 2*, immediately preceding motif 1 [112,113,114,115]. Motif 1 is commonly flanked by basal and distal helices, but recent analysis identified a TURBS that lacks the downstream helical element [116]. The TURBS also interacts with eIF3, which enhances reinitiation [117], possibly by stabilizing the TURBS–ribosome interaction and/or by promoting recruitment of the eIF2-GTP/initiator tRNA ternary complex [118].

Remarkably, analysis of *Megrivirus aturhepa* sequences revealed a 113 nt long TURBS that formed a single extended domain with five helical elements and a canonical UGGGA motif 1 in a centrally located loop that has the potential to base-pair with the apex of helix 26 of 18S rRNA (Figure 4B). With the caveat that this putative megrivirus TURBS has not been experimentally validated, this element is longer than the 40–80 nt long calicivirus TURBS and thus has the potential to interact more extensively with eIF3.

A second group of related megrivirus genomes that contain ORF2 elements that encode FAST proteins is epitomized by *Megrivirus chigalli* (e.g., Megrivirus C2 isolate 27C (NCBI Ref. Seq. NC_024769.1)). It contains a conserved sequence upstream of the conserved stop/start sequence (AUGUAAAUU**AUG**, in which the stop codon is underlined and the initiation codon is bold). However, it lacks a motif 1 (UGGGA) sequence and does not appear to form a conventional TURBS. If this ORF2 is translated, it remains to be determined whether translation results from a divergent termination–reinitiation mechanism or is mediated by an IRES embedded within the 3′-terminal region of ORF1.

## 9. Conclusions

A rapidly expanding number of picornavirus genomes has been found to contain a second ORF in addition to the principal polyprotein-encoding ORF1. ORF2 occurs at various positions: overlapping the 5′-terminal region of ORF1, upstream of ORF1, downstream of it or contained entirely within it. Although several ORF2 candidates reported here reman to be validated, it is notable that many have been found to be subject to strong purifying selection [25], which is suggestive of their functional importance. A systematic bioinformatic search would almost certainly identify additional candidates for experimental verification. In light of the identification of validated and potential altORFs near the 5′ and 3′ extremities of some picornavirus genomes [76,77,78,79,81,82], it is possible that many reported genomic sequences that contain a full polyprotein-encoding ORF may nevertheless be incomplete and that this may have resulted in altORFs having been overlooked.

This diversity in ORF positioning is matched by the diversity of function of the encoded polypeptides. The various second cistrons encode different classes of protein, including the TMEV L* protein (an RNAse L inhibitor) [96], the EMCV 2B* protein (which promotes cell lysis to facilitate viral release and binds to 14-3-3 proteins to impair innate immune signaling [107,108]), and the enterovirus ORF2 product, a putative *trans*-membrane protein that facilitates viral release from cells [7,85]. Several other picornavirus altORFs are predicted to encode *trans*-membrane polypeptides, including megrivirus and poecivirus ORF2-encoded polypeptides that belong to the fusion-associated *trans*-membrane (FAST) class of proteins [78], whereas others are predicted to contain *trans*-membrane domains but lack other characteristics of FAST proteins. The functions of FAST-related and other potential *trans*-membrane proteins in the infection cycle of picornaviruses remain to be determined.

Investigation of picornavirus translation has focused almost exclusively on IRES-mediated initiation, and translation of many of the confirmed and hypothesized ORF2 moieties described here occurs as a result of this process, either due to IRES duplication (which allows initiation to occur at independent sites) or IRES-mediated initiation followed by leaky scanning. Notably, at least three other processes may be responsible for ORF2 translation, namely end-dependent but cap-independent initiation, programmed ribosomal frame-shifting and TURBS-mediated termination–reinitiation. Exploitation of these alternative mechanisms would not only enable ribosomes to gain access to alternate ORFs, but would also allow the level of synthesis of the encoded products and of the timing of their formation to be regulated during the infection cycle.

## Data Availability

All novel data presented in this work are contained within the manuscript figures.

## Abbreviations

The following abbreviations are used in this manuscript:

altORF	alternative open reading frame
ABCE1	ATP-binding cassette subfamily E member 1
CPDV	canine picodicistrovirus
DHX29	DExH-box helicase 29
eEF	eukaryotic elongation factor
eIF	eukaryotic initiation factor
EMCV	encephalomyocarditis virus
eRF	eukaryotic release factor
FAST	fusion-associated small transmembrane
FMDV	Foot-and-mouth disease virus
GDP	Guanosine diphosphate
GTP	Guanosine triphosphate
HAV	Hepatitis A virus
IGR	intergenic region
IRES	Internal ribosomal entry site
ITAF	IRES *trans*-acting factor
NCBI	National Center for Biotechnology Information
ORF	Open reading frame
PCBP2	Poly(rC)-Binding Protein 2
RNA	Ribonucleic acid
RNAse	ribonuclease
RNP	ribonucleoprotein
rRNA	ribosomal RNA
TMEV	Theiler’s murine encephalomyelitis virus
tRNA	transfer RNA
TURBS	termination upstream ribosome binding site
UTR	Untranslated region
VPg	viral protein genome-linked
